# Remote monitoring of pulmonary artery pressures with CardioMEMS in patients with chronic heart failure and NYHA class III: first experiences in the Netherlands

**DOI:** 10.1007/s12471-017-1054-4

**Published:** 2017-11-10

**Authors:** J. J. Brugts, O. C. Manintveld, N. van Mieghem

**Affiliations:** Erasmus MC Thorax Center, Rotterdam, The Netherlands

**Keywords:** Heart failure, CardioMEMS, Telemonotoring, Remote monitoring, Individualized therapy

## Abstract

We report the first patient experiences with the CardioMEMS device in the Erasmus MC Thorax Center in the Netherlands. In line with clinical trial evidence, the device is applicable in patients with chronic heart failure in functional New York Heart Association class III with at least 1 admission for heart failure in the past 12 months. CardioMEMS has been shown to be safe and reliable, and effective in reducing the number of hospitalisations for heart failure by guided therapy based on pulmonary artery pressures.

## Case/patient demonstration

We present a case of a 59-year-old male patient with a history of diabetes mellitus and an anterolateral myocardial infarction in March 2016 for which a percutaneous coronary intervention (PCI) of the left anterior descending artery and left circumflex artery was performed. Initial echocardiogram demonstrated a severely depressed left ventricular function with an ejection fraction of 15% and severe mitral insufficiency. The patient was decongested with diuretics and treated with standard heart failure medication according to guidelines. He developed low cardiac output syndrome with prerenal renal insufficiency with minor changes in angiotensin converting enzyme (ACE) inhibitor dosages and orthostatic symptoms. The balance between stable heart failure, renal insufficiency and adequate dosages of heart failure (HF) treatment was delicate but finally achieved. We performed a Swan-Ganz right heart catheterisation with no pulmonary hypertension and normal pulmonary capillary wedge pressure. At the outpatient clinic, heart failure medications were titrated to maximum tolerance. Due to development of dry cough, we switched Ramipril to valsartan with relieve of cough but prerenal renal insufficiency remained, with an estimated glomerular filtration rate (eGFR) of 35–45. Six months later, follow-up echocardiogram demonstrated a severely depressed left ventricular function and signs of elevated right heart pressures. Due to progressive renal insufficiency, we switched valsartan to hydralazine and intensified isosorbide dinitrate and diuretics. The patient’s functional New York Heart Association (NYHA) class was III and a VO2max was performed with a score of 16 ml/kg/min. At this stage, we admitted the patient to examine the pulmonary hypertension. There were no options to increase diuretics as the patient was euvolemic and suffered from prerenal moderate renal insufficiency. Invasive Swan-Ganz measurement confirmed the estimated pressures on echocardiogram with a pulmonary artery (PA) systolic pressure of 87 and a PA diastolic pressure of 34 (mean PA pressure 52 mm Hg). We decided to offer the patient a new device (CardioMEMS) to monitor his pulmonary pressures remotely with respect to treatment alterations which were previously difficult to achieve based on physical examination, weight and blood values alone.

## Telemonitoring in heart failure patients with the CardioMEMS device

The CardioMEMS device is an implantable wireless system for continuous monitoring of PA pressures which has been shown to be safe and reliable and clinically effective in reducing HF associated hospitalisations in the CHAMPION trial [1]. Complication rate of the implantations are low with 1.4% which is comparable to a regular right heart catheterisation (approved by the Food and Drug Administration and CE (Conformité Européene) marked). The rationale of the device is to use a pressure-driven clinical strategy instead of waiting for the development of weight gain and symptoms of decompensation, which occur generally 1–2 weeks after the rise in left ventricular filling pressures [1]. With progression of heart failure, initially only the filling pressure will increase, leading to autonomic adaptations and changes in intrathoracic impedance. After that stage, patients are retaining fluid and will eventually gain weight and develop symptoms of congestion. This process takes about 2 weeks and diuretics are used to counteract this process, which, if that fails, will lead to HF hospitalisation. The rationale of the CardioMEMS device is to measure those pressures and act on elevated pressures before weight gain and symptoms occur in order to prevent HF hospitalisation.

The CHAMPION trial included patients with chronic heart failure NYHA class III, irrespective of left ventricular ejection fraction and a previous HF hospitalisation. The trial randomised 560 patients to wireless implantable haemodynamic monitoring versus standard care alone. In 6 months, a significant reduction in hospitalisations for HF was demonstrated compared with the control group (hazard ratio 0.63; 95% confidence interval 0.52–0.77; *p*-value < 0.0001). Several centres in the Netherlands have been asked to participate in a post-marketing surveillance programme from Germany. The Erasmus Medical Center has performed multiple procedures, so far successfully, with comparable clinical outcome as presented by the example in this case. CardioMEMS is currently only available in a study environment and not available as standard care in the Netherlands, nor is it covered by medical insurance.

**Fig. 1:**
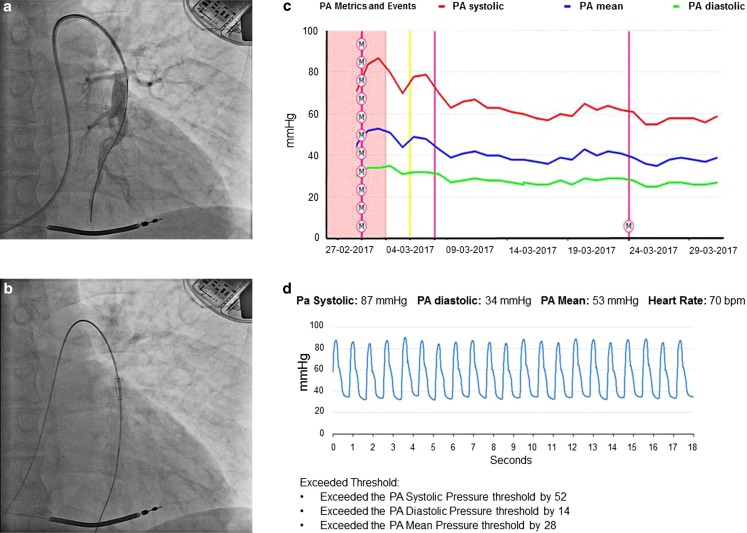
CardioMEMS implantation and data read-outs. **a** Pulmonary arteriography and implantation, **b** placing the CardioMEMS device over the wire with dislodgement, **c** read-out overview of follow-up with the CardioMEMS pressure during medical interventions with daily pressure feedback, **d** example of data read-out: pulmonary pressure read-out

In our patient, the CardioMEMS device was implanted without complications (Fig. 1a and b). The pressure curves of the device were identical to Swan-Ganz. Based on the pressure curves, we titrated the hydralazine dose to 40 mg t.i. d. and isosorbide dinitrate dose to 40 mg t.i. d. With every adjustment in medication, the pulmonary pressures decreased (see the trend curve in Fig. 1c). The patient received instructions and training on how to obtain data from the device and perform daily pressure measurements at home (Fig. 1d). Under the guidance of pressure, the changes in medication were performed with more confidence. Now, after one month of guidance, the patient is feeling well, HF is NYHA class IIb, with current pressures of 55/25 (35) mm Hg and still dropping. No hospitalisations for HF occurred. Renal function is stable with an eGFR of 45–50. The mitral regurgitation decreased from moderate to light. As pulmonary hypertension developed rather quickly in this patient who initially had no elevated pressures, we are curious, but as yet do not known, how quickly he will respond to treatment. However, the decreasing trend is reassuring. We have tested for reversibility of pulmonary hypertension with nitroglycerin at the catheterisation laboratory, which was positive.

For the treating heart failure specialist, the immediate feedback of pressure monitoring is a great step forward. However, it does change the way we work and think at the outpatient clinic. In the current study protocol, the patient performs daily pressure measurements and the cardiologist receives immediate feedback on recorded pressures with notifications based upon set thresholds. Each week, the physician checks the trend of mean PA pressure according to study protocol. The physician can react on the weekly trend of PA pressures by adjusting the medication, such as the dose of diuretics, nitrates or other vasodilators. So, with this new feature of pressure-guided therapy, the physician can provide immediate and truly individualised and tailored therapy, instead of having to rely solely on the information of the patient’s symptoms and weight. The patients report a greater convenience and confidence as they are monitored daily and they feel much more secure now. Surely, an important psychological effect. With more devices being implanted and more experience gained, we must get a better understanding of the role these new technologies will have in heart failure care pathways and rewrite our heart failure clinic set-ups. Currently, the costs of these new technologies are high and their clinical use is limited to participation in post-approval studies. In the near future, when more experience is gained (and the treatment covered by medical insurance in the Netherlands), individualised patient care via telemonitoring can change the clinical practice for physicians, specialist heart failure nurses and, most importantly, patients.
